# Substantial heterogeneity of inflammatory cytokine production and its inhibition by a triple cocktail of toll-like receptor blockers in early sepsis

**DOI:** 10.3389/fimmu.2023.1277033

**Published:** 2023-10-05

**Authors:** Willem Buys, Alexandra Bick, Rabea J. Madel, Astrid M. Westendorf, Jan Buer, Frank Herbstreit, Carsten J. Kirschning, Jürgen Peters

**Affiliations:** ^1^ Universität Duisburg-Essen, Essen, Germany; ^2^ Klinik für Anästhesiologie und Intensivmedizin, Universität Duisburg Essen & Universitätsklinikum Essen, Essen, Germany; ^3^ Institut für Medizinische Mikrobiologie, Universität Duisburg Essen & Universitätsklinikum Essen, Essen, Germany

**Keywords:** whole blood assay, cytokine, early sepsis, hyperinflammation, immune modulation, toll-like receptor, heterogeneity

## Abstract

**Introduction:**

Early sepsis is a life-threatening immune dysregulation believed to feature a “cytokine storm” due to activation of pattern recognition receptors by pathogen and danger associated molecular patterns. However, treatments with single toll-like receptor (TLR) blockers have shown no clinical benefit. We speculated that sepsis patients at the time of diagnosis are heterogeneous in relation to their cytokine production and its potential inhibition by a triple cocktail of TLR blockers. Accordingly, we analyzed inflammatory cytokine production in whole blood assays from early sepsis patients and determined the effects of triple TLR-blockade.

**Methods:**

Whole blood of 51 intensive care patients sampled within 24h of meeting Sepsis-3 criteria was incubated for 6h without or with specific TLR2, 4, and 7/8 stimuli or suspensions of heat-killed *S. aureus* or *E. coli* bacteria as pan-TLR challenges, and also with a combination of monoclonal antibodies against TLR2 and 4 and chloroquine (endosomal TLR inhibition), subsequent to dose optimization. Concentrations of tumor necrosis factor (TNF), Interleukin(IL)-6, IL-8, IL-10, IL-1α and IL-1β were measured (multiplex ELISA) before and after incubation. Samples from 11 sex and age-matched healthy volunteers served as controls and for dose-finding studies.

**Results:**

Only a fraction of sepsis patient samples revealed ongoing cytokine production *ex vivo* despite sampling within 24 h of first meeting Sepsis-3 criteria. In dose finding studies, inhibition of TLR2, 4 and endosomal TLRs reliably suppressed cytokine production to specific TLR agonists and added bacteria. However, inflammatory cytokine production *ex vivo* was only suppressed in the high cytokine producing samples but not in the majority. The suppressive response to TLR-blockade correlated both with intraassay inflammatory cytokine production (r=0.29–0.68; p<0.0001–0.04) and cytokine baseline concentrations (r=0.55; p<0.0001).

**Discussion:**

Upon meeting Sepsis-3 criteria for less than 24 h, a mere quarter of patient samples exhibits a strong inflammatory phenotype, as characterized by increased baseline inflammatory cytokine concentrations and a stark TLR-dependent increase upon further *ex vivo* incubation. Thus, early sepsis patient cohorts as defined by Sepsis-3 criteria are very heterogeneous in regard to inflammation. Accordingly, proper *ex vivo* assays may be useful in septic individuals before embarking on immunomodulatory treatments.

## 1 Introduction

Sepsis is a life-threatening immune dysregulation that damages the host and constitutes a relevant health problem worldwide (1). Early sepsis pathology has been attributed to a “cytokine storm” (2), a self-amplifying signaling loop leading to an immune cell overactivation with the production of biocides and proteases, apoptosis and necroptosis, endothelial damage and cleavage of coagulation proteins, and thus direct (*e.g.*, cell mediated) and secondary (*e.g.*, through microthrombosis) organ damage (3). This “unleashing of the immune system” (4) has been linked to the secretion of inflammatory cytokines in response to stimulation of Toll-like receptors (TLR) by pathogen and danger associated molecular patterns (P/DAMP) (5–8). However, this classic concept is challenged by the observation that in experiments even high dosages of exogenous inflammatory cytokines do not reproduce sepsis or septic shock to a satisfactory degree and inflammatory cytokine concentrations are often surprisingly low in clinical sepsis (9, 10). Furthermore, multiple clinical trials aimed at dampening an overshooting immune reaction have failed. Specifically, blockade of Toll-like receptor (TLR) 2 or 4, blockade of TNF or IL-1 signaling, and attempts to remove IL-6 and other proinflammatory cytokines from the blood all failed to decrease mortality (11–17). In fact, in a phase 3 clinical trial, a TLR4-blocker did not even decrease the concentration of any of the inflammatory cytokines measured including IL-6, TNF, IL-8, and IL-1β (11).

Accordingly, the merit of single TLR-blockade in sepsis stands in question. To interrogate, whether to abandon or refine this concept, *i.e.*, whether TLR-blockade has potential as sepsis therapeutic, three questions should be addressed: 1) Are inflammatory cytokine concentrations universally increased in the hosts` blood in early sepsis, *i.e.*, at a time when the diagnosis of sepsis is made clinically? 2) Does this translate to an ongoing inflammatory activity with cytokine production *ex vivo*? 3) Can this cytokine production be attenuated by a comprehensive inhibitor cocktail blocking sepsis-relevant TLR.

Consequently, we analyzed blood samples of sepsis patients within 24 h of first meeting Sepsis-3 criteria (18) for the presence of inflammatory cytokine concentrations in their blood, for ongoing cytokine production *ex vivo*, and, following dose finding studies, for the effects of a combined (triple) TLR blockade. In addition, we tested the reactivity of patient samples to bacterial stimuli with and without TLR blockade, to validate the system and confirm the activity of the applied blockers in patient samples. We chose blood assays rather than isolated cells as the main experimental model since these contains all elements present in the sepsis patients’ circulation at sampling including their current immune cell composition, danger associated molecular patterns (DAMPs), and possibly also bacteria or their fragments (pathogen associated molecular patterns, PAMPs), as shown previously.

## 2 Materials and methods

### 2.1 Septic patients and controls, clinical data collection, and blood sampling

This prospective observational study, addressing effects of multi Toll-like receptor (TLR) blockade in whole blood *ex vivo* assays is a companion study to a previous one addressing P/DAMP concentrations and effects of immune checkpoint inhibition in sepsis using the same inclusion/exclusion criteria and cohorts (5). Briefly, following ethics committee approval (Medical Faculty, #17-7330-B0), Intensive Care Unit (ICU) patients at the Universitätsklinikum Essen, Germany, were screened for early sepsis using the current Sepsis-3 criteria (18), *i.e.*, showing an increase in the sepsis-related/sequential organ failure assessment (SOFA) score by ≥2 points and suspected or confirmed causative infection. Patients meeting Sepsis-3 criteria for over 24 hours already, those under 18 years of age, on immunosuppressive medication before sepsis onset, or with HIV infection were excluded. Immunosuppressive medication was defined as over 30 mg/d cortisol equivalent (*i.e.*, the Cushing-threshold).

Blood from 51 sepsis patients was drawn via catheters (arterial or central venous, as available) into tubes containing unfractionated heparin (final concentration 16 IU/ml blood; 02.1064, Sarstedt, Nümbrecht, Germany) and transferred to our laboratories at room temperature within 30 minutes. Inflammatory, hemodynamic, and metabolic markers of sepsis patients and derived scores are shown in **Table 1**. Pneumonia was the most common causative infection (n=33), 8 patients had a urinary tract infection, 6 patients had a peritonitis, 3 patients had other foci (endocarditis, arthritis, and a soft tissue infection), 6 patients had multiple plausible infectious foci, and the focus was unclear in 7 cases. Gram-negative bacteria were the most common causative infectious etiology (**Table 1**), but multiple cases of mixed bacterial and fungal or viral co-infections were also recorded. The eventual fatality rate was approximately 45%.

**Table 1:** Characteristics of sepsis patients just meeting sepsis-3 criteria.

	High inflammatory cytokine producers	Low inflammatory cytokine producers	All sepsis patients
Age [years]	53 [42|64]*	65 [53|76]	63 [51|69]
Sex Men [n; %] Women [n; %]	9; 69	22; 58	31; 61
	4; 31	16; 42	20; 39
Heart rate [min^-1^]	83 [75|115]	110 [90|120]	100 [85|120]
Systolic arterial pressure [mmHg]	110 [90|120]	100 [93|120]	105 [90|120]
Diastolic arterial pressure [mmHg]	63 [60|83]	50 [50|60]	50 [40|60]
Mean arterial pressure [mmHg]	67 [63|80]	70 [64|77]	70 [63|80]
Leukocyte concentration [nl^-1^]	16.3 [15.4|20.0]	19.6 [13.0|28.9]	16.6 [13.0|26.2]
Monocyte concentration [µl^-1^]	619 [509|1045]	484 [380|910]	599 [417|975]
Case fatality rate [%]	38	47	45
ICU stay, overall [days]	28 [8|50]*	10 [6|23]	13 [6|28]
ICU stay, survivors [days]	42 [16|64]	12 [7|26]	12 [7|27]
ICU stay, deceased [days]	7 [5|19]	9 [5|17]	13 [6|28]
SOFA-Score [median]	13 [11|15]	12 [11|14]	12 [11|14]
SAPS-II Score [median]	66 [55|78]	73 [60|80]	72 [60|79]
≥2 qSOFA criteria [%]	69	95	86
≥2 SIRS criteria [%]	92	89	90
Shock [%]	69	50	54
Pat. with i.v. catecholamine therapy [%]	69	94	86
Pat. with i.v. catecholamine therapy ≥ 0.1 µg/kg body weight/min [%]	38	62	54
Lactate serum concentration [mmol/l]	2.4 [1.8|4.5]	2.1 [1.6|3.8]	2.2 [1.6|3.9]
Patients on mechanical ventilation (%)	77	82	80
P_a_O_2_/F_i_O_2_ ratio (mmHg)	226 [89|311]	164 [98|222]	172 [96|249]
Platelet concentration [nl^-1^]	98 [61|189]	173 [85|290]	156 [77|258
Bilirubin serum concentration [mg/dl]	1.5 [0.9|1.8]	0.8 [0.4|2.8]	1.0 [0.5|2.3]
Creatinine serum concentration [mg/dl]	1.7 [1.1|2.3]	1.4 [1.0|2.3]	1.6 [1.0|2.3]
C-reactive protein serum concentration [mg/l]	17.5 [11.4|30.3]	16.5 [11.0|23.4]	17.0 [11.1|27.7]
Procalcitonin serum concentration [µg/l]	3.5 [2.0|29.6]	4.3 [1.2|15.5]	4.0 [1.6|15.8]
Gram-positive germ detected (%)	38	23	27
Gram-negative germ detected (%)	53	45	47
Fungus detected (%)	46	7	17
Virus detected (%)	0	5	4

Clinical data and derived scores such as SOFA, qSOFA, SIRS, and SAPS-II score of 51 sepsis patients showing high (n=13) or low (n=38) inflammatory cytokine production in whole blood ex vivo assays. Data are presented as number and percentage or median with upper and lower quartile, as appropriate. Microbiology obtained by PCR-panel and/or culture from blood or primary focus. Mann-Whitney test or Fisher’s exact test, as appropriate. *p<0.05 compared to low producers.

Eleven healthy adults served as age and sex matched controls after ethics committee approval (#17-7869-B0) and written informed consent. While leukocyte concentrations differed between sepsis patients and controls, their monocyte concentrations and hence the major source of inflammatory cytokines did not. As dose finding studies were performed to establish proper concentrations of agonists and antagonists before the main experiments, blood from some volunteers was sampled on multiple occasions. Due to limited available sample volumes especially from sepsis patients, not all experiments could be performed on all samples.

To avoid bias, patient care and laboratory personnel were unaware of all anonymized data. Since neither patients nor volunteers underwent health-related interventions, registration as a clinical study was not applicable as confirmed before enrollment by the German clinical study register.

### 2.2 Whole blood assays

We chose whole blood assays as a common (19, 20) experimental model of septic immunity, as whole blood contains cells of the macrophage family, neutrophils, and lymphocytes, and unlike tissue resident immune cells, is rather easily available *ex vivo*. We chose 6 hours as standard incubation time to limit changes in blood cell composition and other culture artifacts during the assays. Twohundred µl per well of heparinized whole blood were distributed to 96 well polystyrene plates (Nunc 262162, Thermo Fisher Scientific, Waltham, MA). Toll-like receptor (TLR) inhibitors, specific TLR-agonists, or general immune stimulants were added subsequently, and suspensions were incubated for 6 h in standard culture conditions (37°C, humidified atmosphere, 5% CO_2_). To assess assay stability, aliquots were incubated for a total of 22 h with or without lipopolysaccharide Re595 (see below) added after 6 h. Of note, sepsis patients’ whole blood as an *ex vivo* model of inflammation remained reactive to extrinsic TLR stimulation by LPS Re595 for at least 22 hours (**Figure S1**), *i.e.*, far longer than the 6 h incubation period used for the main assays. After incubation, supernatants were retrieved, and mediator concentrations were measured. Individual assays were run in biological triplicates.

### 2.3 Mediator concentrations in blood plasma and whole blood assay supernatants

To reliably measure cytokine concentrations over a wide concentration range, we chose Luminex assays (Luminex LXSAH-06, R&D Systems, Minneapolis, MN) with an improved sensitivity and intertest reliability over standard enzyme-linked immunosorbent assay. In brief, inflammatory immune mediators in blood plasma before and after incubation are adsorbed to antibody-coated, color-coded beads, and the signal is amplified over enzyme-coupled secondary antibodies, before measurements by flow-cytometry. Samples were prepared according to the manufacturer’s instructions and measured against a standard curve with standards provided in the sets. We chose four inflammatory cytokines commonly associated with human sepsis (TNF, IL-6, IL-1α and β), the major granulocyte attractant IL-8 (≙chemokine CXCL8), and IL-10 as an important anti-inflammatory cytokine. We limited our analyses to a set of six cytokines to reflect both “inflammatory” and “anti-inflammatory” cytokines, within economic considerations. The association of these mediators with sepsis immunity and mortality has been demonstrated (10, 21). After incubation, triplicate samples were pooled for Luminex ELISA.

For pilot experiments, we chose TNF and IL-6 as the most often used and clinically established sepsis-related cytokines. Here, cytokine concentrations were measured by standard colorimetric ELISA (DY206 & DY210, R&D Systems, Minneapolis, MN) against a standard curve according to the manufacturer’s instructions and with standards provided in the sets. Triplicate samples were measured individually with standard ELISA.

### 2.4 Pattern recognition receptor challenges of whole blood samples and effects of TLR-inhibitors: dose-response studies

**Figure 1:**
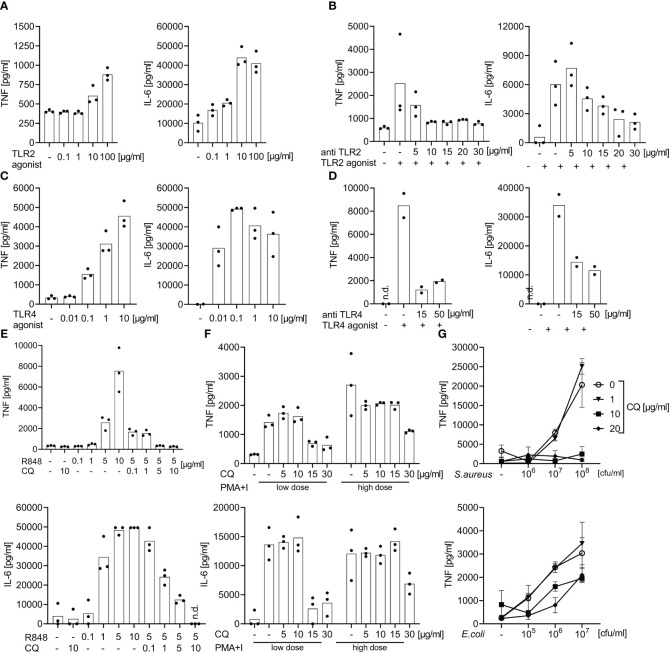
Effect of specific TLR2, 4, and 7/8 agonist stimulation or of bacterial immune stimulation with and without TLR blockade: dose finding studies for TLR blockade. Supernatant TNF and IL-6 concentrations measured by ELISA after 6 h of incubation of whole blood from healthy volunteers following stimulation with TLR2, 4, or 7/8 agonists or with heat-killed bacteria with and without antibodies directed against TLR2, TLR4, or with chloroquine. Concentrations as indicated. **(A)** Effects of the TLR2 agonist P_3_C. **(B)** Addition of anti-TLR2 mAb T2.5 30 min prior to the addition of the TLR2 agonist P_3_C (10 µg/ml). **(C)** Effects of the TLR4 agonist LPS : Re595. **(D)** Addition of anti-TLR4 mAb 3C3 30 min prior to addition of TLR4 agonist LPS : Re595 (0.1 µg/ml). **(E)** Effects of the endosomal inhibitor chloroquine (CQ) 30 min prior to the addition of TLR7/8 agonist Resiquimod (R848). **(F)** Addition of the endosomal inhibitor chloroquine (CQ) 30 min prior to addition of the TLR-independent immune stimuli phorbol-12-myristate-13-acetate and Ionomycin (PMA+I) as a toxicity control for CQ; low dose of PMA+I is 0.2 + 0.14 µg/ml, high dose is 1 + 0.7 µg/ml. **(G)** Addition of the endosomal inhibitor chloroquine (CQ) 30 min prior to the addition of heat-killed *S. aureus* or *E*. *coli*. n.d., not detected. Measurements from 2-3 individuals each. **(A–F)** single-point graph with means, **(G)** means with standard deviation.

Prior studies have established TLR2 and TLR8 as crucial for the recognition of Gram-positive bacteria, and TLR4 and TLR8 for Gram-negative bacteria, and also have established appropriate inhibitors for their use in cell cultures and animals (22–24). To assess the ability of a cocktail of TLR antagonists to block a TLR-dependent cytokine production in whole blood, doses were established in pilot studies using healthy volunteers’ samples (**Figure 1**). In whole blood samples from sepsis patients and controls these agonists were then titrated against monoclonal antibodies (mAb) against TLR2 and 4 and against chloroquine, a drug known to suppress endosomal TLR activity (25).

Specifically, the following TLR-ligands/stimulants were used as immune stimuli in whole blood assays: P_3_C [bacterial lipoprotein mimicking synthetic lipohexapeptide (26)], a TLR2 stimulant (EMC microcollections, Tübingen, Germany), lipopolysaccharide [LPS; *Salmonella minnesota* Re595 (27)], a LPS devoid of a long O-chain, TLR4 specific (Sigma-Aldrich, St. Louis, MO), R848 [synthetic nucleic acid analogue (28)], a TLR 7/8 stimulant (Invivogen, Toulouse, France), sterile PBS suspensions of *S. aureus* (29) (Deutsche Sammlung von Mikroorganismen (DSM)20231, Braunschweig, Germany), and *E. coli* [clinical isolate 30/185 (22)], inactivated at 96° C for 15 minutes. We chose Gram-positive *S. aureus* and Gram-negative *E. coli* as prototypical sepsis-related pathogens. The calcium ionophor ionomycin in combination with the protein kinase C agonist phorbol-12-myristate-13-acetate [PMA (30)] were used as TLR-independent stimuli of cytokine production and as a toxicity and specificity control for chloroquine.

For TLR blockade we used the anti-TLR monoclonal antibodies (mAb) T2.5 ( (23), TLR2 antagonist; Invivogen, Toulouse, France) and 3C3 ( (31), TLR4 antagonist; Hycult Biotech, Uden, Netherlands), as well as the small molecule antimalarial and endosomal TLR-inhibitor chloroquine ( (25), Sigma-Aldrich, St. Louis, MO). In dose finding experiments, these inhibitors were added 30 minutes prior to the addition of TLR-stimuli. Chloroquine is a well-established anti-malarial and has seen some use as anti-inflammatory drug in acute, as well as chronic inflammation (32). In previous studies, we had demonstrated chloroquine’s effectiveness against septic inflammation through its endosomal TLR inhibition (24). In addition, we demonstrated its specificity and non-toxicity (in the chosen doses) by titration against the non-TLR immune stimuli ionomycin and PMA (see above and **Figure 1**). We titrated the TLR-inhibitors against the first concentration of TLR-stimuli that elicited a saturated/plateau cytokine response. Two to three healthy volunteers’ samples were included per dose finding experiment.

### 2.5 Bacterial challenges of whole blood samples and effects of TLR-inhibitors: validation in patients’ samples and controls

**Figure 2:**
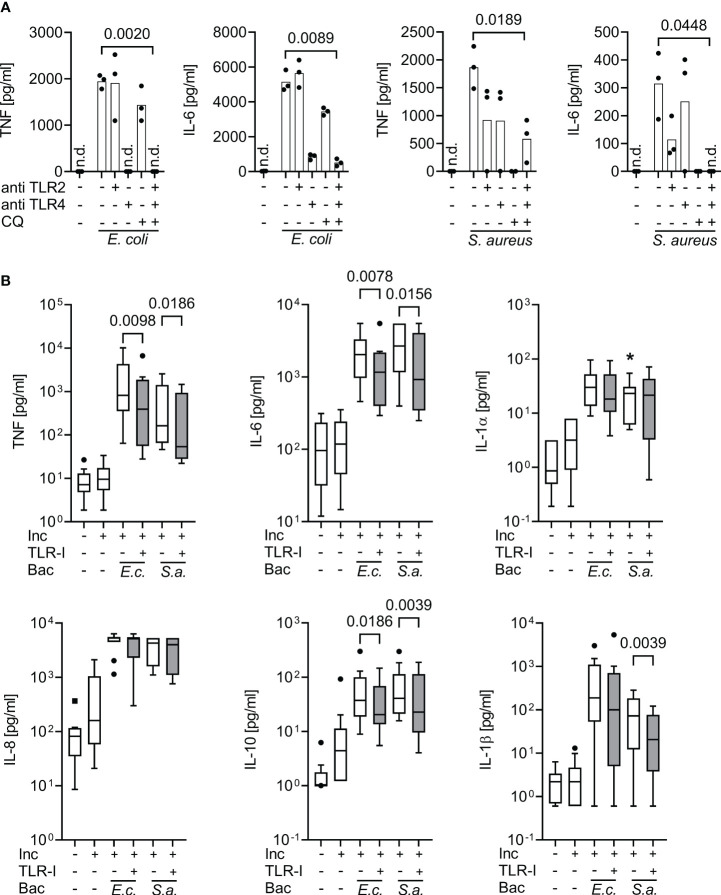
Inhibition of supernatant inflammatory cytokine production by triple Toll-like receptor blockade upon bacterial stimulation in whole blood assays. Cytokine supernatant concentrations (ELISA) before and after incubation for 6 h of whole blood without or with heat-killed *E. coli* (10^5^ cfu/ml; E.c.) or *S. aureus* bacteria (10^6^ cfu/ml; S.a.) and without or with TLR blockade by 15 µg/ml monoclonal antibodies T2.5 and 3C3 directed against TLR2 (anti TLR2) and TLR4 (anti TLR4), or with 10 µg/ml chloroquine (CQ), or with a triple combination (TLR-I) when added 30 min prior to addition of bacterial stimuli. **(A)** Healthy volunteers (n=3). Single-point graph with means. Student’s t-test for paired samples. **(B)** Sepsis patients sampled within 24 h of sepsis diagnosis (n=10), multiplex (Luminex) ELISA. Tukey Boxplots. Wilcoxon matched pairs test. n.d., not detected. *one data point out of axis limits. Inc, Incubation for 6 h; Bac, added Bacteria. A combined blockade of TLR2, 4, and endosomal TLRs effectively suppresses bacteria-stimulated inflammatory cytokine production in whole blood assays both from healthy volunteers and sepsis patients.

The dose finding experiments established a concentration of 15 µg/ml for both TLR inhibitory antibodies and of 10 µg/ml for chloroquine, as appropriate for inhibition of TLR2, 4 and endosomal TLR agonist activity in human whole blood. For validation, the inhibitor combination in the established concentrations was added to healthy volunteers’ whole blood and to the whole blood of 11 sepsis patients 30 minutes prior to adding *S. aureus* or *E. coli* (**Figure 2**).

### 2.6 Cytokine production in whole blood from sepsis patients and controls without and with triple TLR blockade

Whole blood of sepsis patients and controls was incubated for 6 hours without or with a triple combination of the TLR-inhibitory mAbs against TLR2 and 4 (T2.5 and 3C3, see above) and chloroquine as endosomal TLR inhibitor in the dosages established in pilot experiments. Supernatant cytokine concentrations were assessed by Luminex assays before and after incubation for 6 hours.

### 2.7 Statistical methods

Figures were designed and statistical analysis was performed using Graph Pad Prism (GraphPad Software, V8.4.3, San Diego, CA). Continuous clinical variables are presented as median with quartiles, discrete clinical variables as percentage of the respective cohort or median with quartiles, as appropriate. Hypotheses were set *a priori*, and statistical tests were two-tailed. Data were analyzed for non-normality using the Shapiro-Wilk test. Non-normally distributed datasets are presented as Tukey boxplots or median with quartiles. To increase robustness against sampling effects and random outliers, we chose to consequently use non-parametric hypothesis tests for patients’ experimental data, despite some reduction in statistical power. This includes the Mann-Whitney U test for unpaired and the Wilcoxon matched pairs signed rank test for paired data, as well as Friedman’s test with Dunn’s post-test corrected for multiple testing (Graph Pad Prism Standard Settings). For pilot assays, Student’s t-tests for paired or unpaired datasets were used, as appropriate.

Spearman’s rank-correlation coefficient (r) of changes in cytokine concentrations associated with incubation and addition of TLR-Inhibitors as well as of baseline concentrations with concentration changes due to TLR-Inhibitors were calculated using Prism standard settings, with the respective 95%-confidence interval and p-values in regard to the null-hypothesis (r=0). Additionally, the effects of the triple TLR-blockade on the change in cytokine concentrations before vs. after incubation are expressed as percentage.

Rank-sums were used to non-parametrically average baseline cytokine concentrations, *ex vivo* cytokine production, and the TLR-I effect over the five proinflammatory immune mediators (*i.e.*, TNF, IL-6, IL-8, IL-1α, IL-1β). In brief, data points within one dataset were ranked from lowest to highest value of each cytokine concentration and ranks were summed over multiple datasets of one dataset family. The samples were individually ranked by their cytokine concentration of each of the five proinflammatory immune mediators (five datasets) measured at baseline or after incubation with or without TLR-inhibitors (3 dataset families), and the five ranks within one dataset family were summed to obtain a value incorporating all five mediators. To assess whether this ranking was valid, *i.e.*, whether strong production of one cytokine correlated with strong production of another cytokine, we calculated a Spearman correlation of the individual cytokine rankings against each other, as well as against the rank-sum (r=0.60-0.73, p<0.0001, **Data Table S1**). In addition, we used the matching efficiency across rows, a quality control parameter of repeated-measure ANOVA’s, to assess homogeneity of ranks across different cytokines (R^2 ^= 0.44, F=3.23, p<0.0001). The patients’ ranks were highly and significantly stable across the five proinflammatory immune mediators, *i.e.*, strong producers of one cytokine also scored high ranks in other inflammatory cytokines and vice versa. We conclude that cytokine production is indeed significantly correlated across cytokines within the same sample and that the production of different cytokines in individual samples can be averaged using rank-sums to synthesize a meaningful overall ranking of cytokine production. For the baseline concentration (matching efficiency R^2 ^= 0.48, F=3.7, p<0.0001; Spearman’s r 0.63 – 0.74, p<0.0001) and the strength of inhibition by TLR-Inhibitors (matching efficiency R^2 ^= 0.56, F=5.05, p<0.0001; Spearman’s r 0.65 – 0.79, p<0.0001), correlation of ranks across cytokines was even stronger (also see **Data Table S1**).

Receiver-operator characteristics were prepared and the 95%-confidence interval (with a p-value regarding the null-hypothesis: area under the curve (AUC)=0.5 were calculated for the prediction whether a patient’s sample was highly reactive to the combined TLR-blockade (*i.e.*, belonging to the fourth quartile regarding the TLR blockers’ effect) based on the paired baseline proinflammatory cytokine concentrations. Spearman’s rank correlation was performed both based on single cytokines as well as on the rank-sum average.

## 3 Results

### 3.1 Heterogeneity of supernatant inflammatory cytokine concentrations in whole blood assays from early sepsis patients

**Figure 3:**
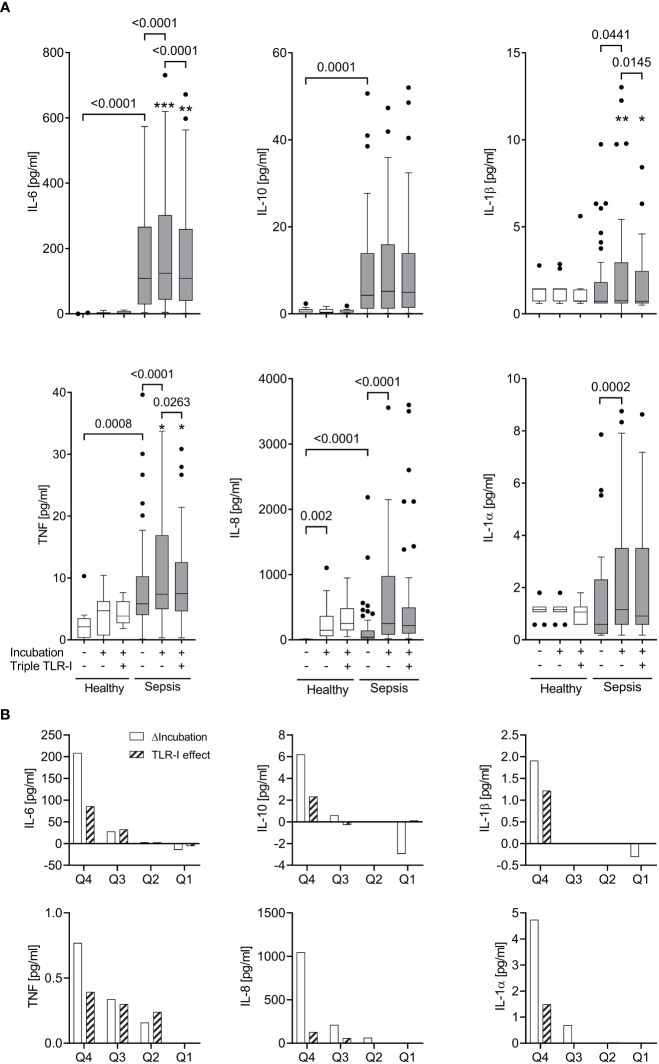
Robust TLR-dependent inflammation only in approximately a quarter of sepsis patients. **(A)** Supernatant inflammatory cytokine concentrations (multiplex ELISA) before and after a 6 h incubation (“Incubation”) of whole blood from sepsis patients (n=51, shaded columns) and healthy volunteers (n=11, open columns) with or without a triple combination of TLR inhibitors (“Triple TLR-I”; anti-TLR2 mAb T2.5, anti-TLR4 mAb 3C3 (both 15 µg/ml), chloroquine 10 µg/ml). Tukey boxplots. Mann-Whitney test for unpaired and Wilcoxon matched-pairs test for paired data sets. *one data point out of axis limits. **(B)** Patient samples sorted by their values for intrinsic *ex vivo* production of IL-6, TNF, IL-10, IL-1α or β over 6 hours (ΔIncubation). Shown are the quartile-medians of ΔIncubation and the paired TLR-I effect (sorted after ΔIncubation). Values from 51 sepsis patients sorted into 4 quartiles.

When incubated for 6 hours without extrinsic stimuli, whole blood from sepsis patients, which already contained greater inflammatory cytokine concentrations than that of healthy volunteers (**Figures 3**, **S2**), showed a significant further increase of supernatant TNF, IL-6, IL-8, IL-1α, and IL-1β concentrations (**Figure 3A**). When stratifying the data sets with regard to supernatant inflammatory cytokine concentrations before and after incubation, however, only about a quarter of the sepsis patients’ samples showed a substantial increase of cytokine concentrations during further incubation. This observation was surprisingly stable across the five proinflammatory cytokines measured (**Figure 3B**, also see ‘statistical methods’).

The patients whose samples showed a strong further increase in inflammatory cytokine concentrations upon incubation (quartile 4 in regard to cytokine production *ex vivo*; “high-producers”) were significantly younger (median 53 | 65 years, p=0.0351) and had a longer ICU stay (median 28 | 10 days, p=0.0498) than the “low-producers” (quartile 1-3; also see **Table S1**). Other variables like SAPS or SOFA score, catecholamine dosage, markers of infection like Procalcitonin or white cell counts, or the type of infection did not reveal any association with *ex vivo* inflammatory cytokine production.

### 3.2 Dose finding studies of TLR-inhibitors against specific TLR agonists in whole blood assays

During a 6 h incubation, P_3_C (TLR2 ligand), bacterial Lipopolysaccharide Re595 (LPS, TLR4 ligand), and R848 (TLR7/8 ligand) evoked positively dose-dependent supernatant concentrations of tumor necrosis factor (TNF) and Interleukin (IL-) 6, reaching supernatant cytokine concentrations far higher than those observed in human sepsis (**Figures 1**, **S2**). A plateau of IL-6 concentrations was reached with 10 µg/ml P_3_C, 0.1 µg/ml LPS, and 5 µg/ml R848, and greater TLR-agonist concentrations did not elicit greater IL-6 concentrations.

At the latter TLR agonist concentrations, supernatant cytokine production was attenuated in a dose-dependent manner by the corresponding TLR-antagonists against TLR2 (mAb T2.5), 4 (mAb 3C3), and endosomal TLRs (chloroquine). Doses of 15 µg/ml for the mAbs and of 10 µg/ml for chloroquine substantially inhibited the stimulated cytokine production and the effect did not substantially increase with even greater blocker concentrations (**Figures 1B, D, E**). While a high concentration of 30 µg/ml chloroquine also suppressed cytokine production evoked by the TLR-independent immune stimuli PMA + Ionomycin, a concentration of 10 µg/ml chloroquine did not decrease the TLR-independent cytokine production (**Figure 1F**) but was effective against TLR-dependent stimuli.

Accordingly, monoclonal antibodies directed against TLR2 and TLR4 combined with chloroquine as endosomal TLR-inhibitor block the *ex vivo* cytokine production towards specific TLR-agonists even at plateau concentrations. Chloroquine alone in a non-toxic concentration inhibits the cytokine production upon immune stimulation with *S. aureus* in whole blood assays. The materials and the TLR-inhibitors themselves did not spark the production of cytokines without the addition of extrinsic stimuli.

### 3.3 Validation of triple TLR inhibition against bacterial stimulation of cytokine production in whole blood assays of volunteers and sepsis patients

At their effective concentrations, the cocktail of TLR-inhibitors blocked the cytokine productions evoked by both, Gram-positive and Gram-negative bacteria (**Figure 2**). In sepsis patients’ whole blood assays, the blockers significantly decreased supernatant concentrations of TNF and IL-6 in response to added heat-killed *E. coli* and *S. aureus*, and of IL-1β in response to *S. aureus*. Interestingly, even the supernatant concentration of the anti-inflammatory cytokine IL-10 was significantly decreased by the combined TLR-blockade.

Accordingly, the TLR blockers mitigated supernatant inflammatory cytokine concentrations even at a maximum/plateau immune stimulation, which was far beyond the inflammatory cytokine concentrations found in our sepsis patient cohort (also see **Figures 1**, **S2**).

### 3.4 Significant correlation of blood-intrinsic *ex vivo* cytokine production with effective TLR inhibition

Overall, the unstimulated increase of inflammatory cytokine concentrations upon *ex vivo* incubation was significantly attenuated by a combined TLR blockade (**Figure 3A**). However, even at a dose that had effectively suppressed far greater cytokine concentrations produced after bacterial stimulation (**Figure 2**), only the IL-6 production was fully suppressed, while the other cytokines were affected to a lesser degree.

**Figure 4:**
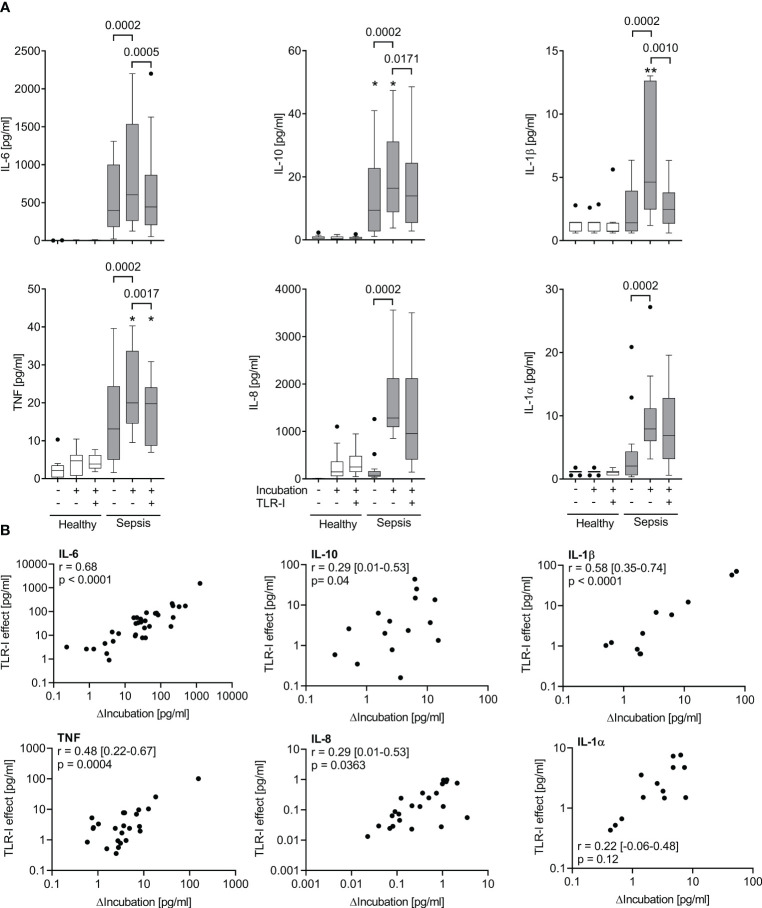
The effect of a combined TLR-blockade is positively correlated with the intrinsic cytokine production *ex vivo*. **(A)** Supernatant inflammatory cytokine concentrations before and after a 6 h incubation of whole blood of sepsis patients (shaded columns) showing the greatest *ex vivo* production (ΔIncubation) of each cytokine after incubation for 6 h (quartile 4 of ΔIncubation) and of healthy volunteers (n=11, open columns) with or without a cocktail of TLR inhibitors (anti-TLR2 mAb T2.5, anti-TLR4 mAb 3C3 (both 15 µg/ml), and chloroquine 10 µg/ml). Data from quartile 4 (n=13 patients). Tukey boxplots. Mann-Whitney test for unpaired and Wilcoxon matched-pairs test for paired data sets. *one datapoint out of axis limits. **(B)** Plot and Spearman’s rank correlation of concentration changes over 6 hours of incubation (ΔIncubation) alone and with added TLR-inhibitors (TLR-I effect). n=51 patients. Negative values cannot be plotted on the logarithmic axes but are included in the analysis. Combined TLR-inhibition strongly inhibits cytokine production in those samples with a high intrinsic cytokine production. Intrinsic cytokine production and TLR-I effect correlate significantly.

When assessing the effect of triple TLR blockade with regard to the samples’ inflammatory cytokine production *ex vivo*, we observed a significant correlation (**Figures 3B**, **4**). Specifically, within the group of high inflammatory cytokine producers, the combined TLR-blockade virtually abolished the *ex vivo* concentration increase of TNF, IL-6, IL-1α and β (*i.e.*, median of differences ≥100%), and decreased it by 78% for IL-8 (absolute values are provided in **Figure 3B**). For the “low-producers”, the TLR blockade reduced the increase of TNF, IL-8, and IL-1α by a maximum of 34% while IL-6 concentrations were still diminished by 54%.

### 3.5 Significant correlation of baseline cytokine concentrations with effect of triple TLR inhibition

**Figure 5:**
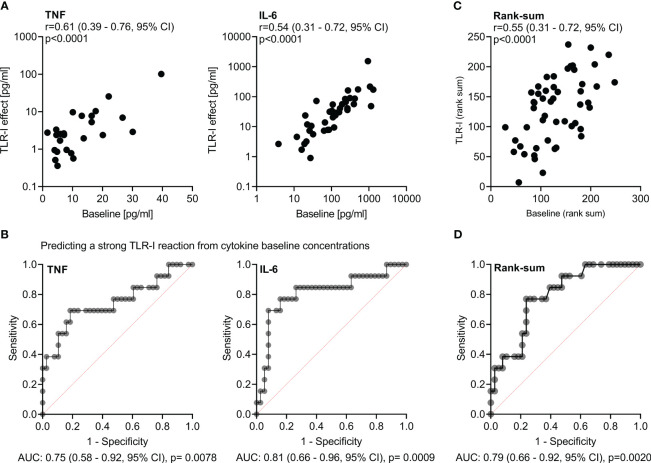
Effects of triple TLR-blockade are correlated with and predicted by baseline IL-6 and TNF concentrations in host plasma. **(A)** Plot and Spearman’s rank correlations of baseline TNF and IL-6 concentrations in host plasma with the effect of triple toll-like receptor inhibition (TLR-I) on cytokine concentration changes over incubation *ex vivo* (expressed as difference in respective concentrations without and with triple inhibition by TLR2, 4, and endosomal TLRs). **(B)** Receiver operating characteristics (ROC), area under the curve (AUC), respective confidence intervals and p-values (regarding the null-hypothesis of AUC=0.5) of the prediction of a strong reaction to TLR-I (*i.e.*, of a patient being in quartile 4 of TLR-I regarding that cytokine) by the baseline concentration of TNF and IL-6. **(C)** Plot and Spearman’s correlation of proinflammatory cytokine concentrations at baseline and corresponding TLR-I effect; averaged via rank-sums over the proinflammatory immune mediators TNF, IL-6, IL-8, IL1α, IL1β. **(D)** Receiver operator characteristic for the prediction of a strong reaction to TLR-I (*i.e.*, being within quartile 4 regarding the TLR-I effect) from the corresponding baseline cytokine concentrations; averaged via rank-sums over the five proinflammatory mediators TNF, IL-6, IL-8, IL-1α, β.

The response to triple TLR blockade *in vitro* also significantly correlated with the baseline proinflammatory cytokine concentrations in sepsis patients` host plasma (r=0.55; 0.31 – 0.72, 95% CI; p<0.0001; **Figure 5**). The AUROC for predicting whether a patient’s sample was attributable to the top quartile regarding their reaction to TLR blockade (*i.e.*, strong blockade) based on their baseline cytokine concentration was 0.79 (0.66 – 0.92, 95% CI; p=0.002). This correlation and prediction were strongest for TNF and IL-6 (**Figure 5**).

## 4 Discussion

Sepsis is generally assumed to be associated with ongoing inflammatory cytokine secretion at least in its initial stages when the diagnosis is made. However, upon meeting Sepsis-3 criteria for less than 24 hours, only a quarter of our patient samples exhibited a strong inflammatory phenotype characterized by increased baseline inflammatory cytokine concentrations and a stark increase upon *ex vivo* incubation. It was also only in this subset of samples, that inflammatory cytokine production was significantly albeit incompletely inhibited by combined triple TLR-blockade. Thus, early sepsis patients are biologically very heterogeneous despite diagnosis and recruitment by the current Sepsis-3 criteria and strict sampling within 24 h of diagnosis. This may explain why prior treatment efforts of dampening inflammation by interfering with the TLR-system have failed overall when administered to an unselected sepsis patient cohort.

In early sepsis, we and others (5, 7, 20, 21, 33) have observed a significant increase of cytokine concentrations upon incubation in *ex vivo* assays and have linked this to increased concentrations of clinically relevant P/DAMPs in host blood, like bacterial and mitochondrial DNA, and TLR2 ligands. Surprisingly, it appears less well investigated whether increased cytokine concentrations in host blood and cytokine production *ex vivo* are uniform features when the clinical sepsis diagnosis is made along current sepsis-3 criteria. Indeed, others have reported surprisingly low average inflammatory cytokine concentrations, especially of the traditional sepsis cytokine TNF, in sepsis cohorts following the sepsis-3 definition (10). Here, we demonstrate in whole blood assays that a strong cytokine production occurs in only a quarter of patients whereas the rest of the samples reveal no such “hyperinflammation”. *Post-hoc* analyses using variables such as kind of infection, SOFA scores, and many others offered no clue as to why some patients` samples reacted so much stronger than others, despite a small but significant age disparity and a longer ICU stay in the high cytokine producing group, possibly suggesting a stronger inflammation. Leukocytes and specifically monocytes, which are the main producers of the cytokines measured, as well as relevant P/DAMP-concentrations (5) which might have driven the *ex vivo* increase also did not differ significantly. Accordingly, other issues or immune phenomena likely play a more important role.

Both in unstimulated and TLR-agonist stimulated whole blood assays of early sepsis patient samples, addition of a cocktail of TLR blockers to the assays significantly and substantially decreased the observed increase of inflammatory cytokine concentrations although only IL-6 was completely suppressed. However, not unexpectedly, this significant decrease of inflammatory cytokine concentrations by triple TLR-blocker application occurred in the same subgroup of patient samples that had shown greater inflammatory activity, *i.e.*, in the high inflammatory cytokine producers. Here, the decrease of supernatant cytokine concentrations by a combined TLR-blockade (by 78-100%) was comparable to the effect of targeted TLR-blockade (34, 35) against its corresponding agonist stimulus both in mice and humans (≈95% reduction). It even surpassed the effect of singular and dual TLR blockade against bacterial infection in mice [25-90% decrease in cytokine concentrations (22–24)]. Triple TLR-inhibition can hence be viewed as effective in this subset of patients showing ongoing inflammatory cytokine production in *ex vivo* assays.

For the whole patient cohort, however, only the increase in IL-6 concentrations was abolished by triple TLR-inhibition (>100% median reduction) while the other cytokines were not or much less affected (≤29% median reduction). Due to its important role in murine sepsis, TNF is put forth by some as the key cytokine in sepsis (9, 10, 36, 37). However, it is found at rather low concentrations in more recent sepsis-3 cohorts (10, 21), whereas IL-6 appears to be more closely correlated with sepsis pathology and has been thoroughly linked to prognosis (38, 39). After IL-6 receptor blockers had successfully been applied against COVID-19 induced hyperinflammation (40), a recent large register study found a correlation of some IL-6 receptor mutations with a lower sepsis incidence and a better survival (41). This may suggest a possible generalization to septic hyperinflammation of IL-6 receptor blockade, although others fear a dangerous increase in secondary infections (42). In preclinical studies, IL-6 production and concomitant TNF-suppression have also been linked to the sepsis-typical co-stimulation of endosomal and cell surface toll-like receptors. This may explain the profound blocker effect on IL-6 as observed in the present study, although other cytokine concentrations were also significantly diminished.

That triple TLR blockade only partially mitigated *ex vivo* cytokine production despite the respective TLR-blockers’ efficacy against added TLR-specific agonists and bacteria as more complex TLR stimuli, may be explained by multiple reasons. First, the monoclonal antibodies used to block TLR receptors could have had a short effective duration. However, in human blood, an effective duration of about 24 hours (43) is expected for mouse IgG, like the ones used in this study, as mouse-antibody Fc-parts are not compatible with the human Brambell receptor (which facilitates IgG recycling and strongly delays antibody clearance). Since this is much longer than our 6 hour incubation time, it is however unlikely that the monoclonal antibody concentrations decreased below a critical threshold during *ex vivo* incubation. In fact, the antibody effect did not subside to a relevant degree in pilot experiments during 6 hours of incubation, where blockers were highly effective against their corresponding specific TLR agonists and against sepsis-relevant bacterial stimuli up to a maximum of evoked cytokine concentrations by far surpassing those measured in our sepsis patient cohort. Specifically, the greatest concentrations for TNF, IL-6, IL-8, and IL-1α measured in unstimulated patient blood were less than even the median concentrations measured in patient blood after bacterial stimulation, at which the blockers were still effective. It is hence very unlikely that greater antibody concentrations would have yielded a greater effect. The same is true for chloroquine, which in dose finding studies suppressed the reaction to Gram-positive bacteria even when applied without monoclonal antibodies while not impeding cellular functionality. This is supported by the unaltered cellular responses to ionomycin and PMA indicating preserved calcium gradients over organelle membranes as well as intact protein kinase C signaling, cytokine gene transcription, translation, and exocytosis.

Accordingly, we observed an ongoing and presumably relevant cytokine production during unstimulated *ex vivo* incubation despite adequate blockade to suppress inflammatory cytokine production in response to TLR stimulation. Thus, independent of the blocked TLRs, other inflammatory pathways might have partially contributed to the sustained inflammatory cytokine production *ex vivo*. For instance, despite the prominent role of the TLRs, there is a multitude of other cell surface (44, 45) and cytosolic (46) pattern-recognition receptors, such as the classic inflammasome to detect Gram-positive and Gram-negative bacterial cell wall components and the non-canonical inflammasome to detect LPS independently of TLRs (47–49). In addition to the recognition of PAMPs, a multitude of DAMPs activate immune cells in sepsis (50). While some of these are recognized via the toll-like receptors inhibited in this study, such as high mobility group box (HMGB)1 via TLR4, cellular RNA via TLR7, 8, and possibly TLR3 (51, 52), and mitochondrial and genomic DNA via TLR9 (25, 53), DAMPs are also recognized via non-TLR pattern-recognition receptors (46). In fact, we had found more significant and more pronounced differences in DAMP than in PAMP concentrations between sepsis patients and healthy volunteers in a companion study (5). DAMPs might accordingly have driven some *ex vivo* cytokine production refractory to triple TLR-blockade in samples from sepsis patients, whereas these mediators were absent in our dose finding studies.

Previous *in vitro* and animal studies had pointed to TLR2, 4, and endosomal TLRs as crucial receptors of early septic inflammation (22–24, 54, 55) and suggested triple blockade for maximum efficacy. Indeed, only a triple combination blocked the cytokine production to both Gram-positive and Gram-negative bacteria in dose finding studies, and they strongly diminished bacteria-stimulated inflammation in sepsis patients’ blood. Nevertheless, this effect failed to translate to the observed blood-intrinsic inflammation in the majority of patients’ samples. Accordingly, while septic inflammation of the blood compartment appears partly dependent on continued TLR2, 4, and endosomal TLR activation by corresponding PAMPs (5), even a combined blockade is not sufficient to abrogate septic hyperinflammation in the whole patient cohort. Rather, some proinflammatory cytokine production is upheld by other mechanisms like TLR-independent stimulatory pathways and cellular programming. This raises the question, whether enough proinflammatory pathways can ever reliably be blocked on the receptor level for a comprehensive effect in a diverse sepsis patient population.

In any case, our data show that sepsis patients at the time of diagnosis by Sepsis-3 criteria, are very heterogeneous with regard to inflammatory cytokine production *ex vivo* and that only a quarter of those is potentially amenable to suppression of inflammatory cytokine secretion even by a combined triple TLR-blockade. This finding also may help to explain why efforts in sepsis patients to dampen hyperinflammation by singular toll-like receptor blockers using small molecules (11) or monoclonal antibodies (14) have failed so far.

Another point to consider is early septic hypoinflammation or endogenous immunosuppression. Indeed, using similar assays in a previous study (5), many sepsis patients’ samples reacted to a lesser extent to added pattern-recognition receptor agonists than samples from healthy volunteers. This might also explain a (non-significant) tendency for a better hospital survival in the high cytokine producer subgroup, as the lack of TLR-dependent cytokine production in the low-producer subgroup could be understood as early hypoinflammation. All this indicates not only an important dispersal of early sepsis phenotypes despite their common definition and timepoint of just meeting Sepsis-3 criteria, but also advocates for individualized sepsis therapy, possibly even requiring novel diagnostic criteria to accommodate for the range of different immunologic sepsis phenotypes. Despite the sepsis definition as a “dysregulated host response to infection” (18), the sepsis-3 criteria focus on organ failure without any immunologic assessment. While certainly a clinically discernable anchor in time for sepsis diagnosis and well validated for predicting mortality in intensive care units (56, 57), this focus on organ failure might still delay recognition (58) and shorten the clinical window for applying proper early sepsis therapies. In fact, secondary analyses of clinical studies have suggested a benefit of anti-inflammatory treatments for those patients who display hyperinflammation at sepsis recognition. Specifically, a *post-hoc* analysis by Shakoory et al. (59, 60) suggests a benefit of IL-1 receptor blockade only for sepsis patients with septic macrophage activation like syndrome. Our observations are also consistent with a large register sepsis study that retrospectively characterized 4 phenotype clusters of sepsis, one of which (about 13% of patients) exhibited significantly greater cytokine concentrations than the others (61). Together, these data advocate for preselection of patients by *ex vivo* assays like the ones used in our study before commencing specific targeted sepsis treatments. In our study, the response to a combined TLR blockade within assays also strongly correlated both with the proinflammatory cytokine concentrations at baseline in host blood and with the further concentration increase upon *ex vivo* incubation. The reactivity to a combined TLR blockade hence indicates a strong proinflammatory phenotype. Preselection for immune therapies by mere cytokine concentrations in host blood has previously failed (16, 17). However, more functional assessments, similar to the ones presented in this study, might provide a more holistic view and improve preselection of future patients for specific treatments rather than applying treatments broadly to the whole and diverse sepsis cohort.

### 4.1 Limitations

Although whole blood assays incorporating all elements of blood, including P/DAMPs, and the host’s responses might be preferable to more reduced experimental systems or isolated cells, such assays cannot completely simulate the complex interplay with other organs and tissue immune cells, which during sepsis might behave differently from blood immune cells. Indeed, tissue resident immune cells of multiple compartments have been reported to show a stronger proinflammatory reaction than blood immune cells (62) and, therefore, an *in vivo* effect of the blockers used could be more pronounced or comprehensive. Furthermore, while we extensively established the TLR-blockers in their ability to suppress the response to established TLR agonists and heat-killed bacteria prototypical for sepsis in both volunteers’ and patients` blood, real sepsis likely involves more P/DAMPs acting on many other targets and pattern recognition receptors, as outlined above, accounting for remaining inflammatory activity.

Sepsis is a highly dynamic disease, and the changes of cytokine concentrations over time have been extensively reviewed elsewhere (21). We hence strictly adhered to the 24 hours timeframe after sepsis diagnosis for study inclusion, although naturally, we cannot rule varying presentations and time scales before sepsis onset as potential sources of variance. To minimize potential changes in blood composition, we limited the assay time to 6 hours. In fact, we demonstrated reactivity of the patients’ whole blood to LPS for at least 22 hours indicating that the absence of substantial cytokine production in many assays was not related to assay conditions or time. Finally, as a tertiary referral center, we cannot exclude patient selection bias and that different results might be observed in less severe sepsis or in other cohorts.

### 4.2 Conclusion

Surprisingly, within 24 hours of just meeting Sepsis-3 criteria, only a quarter of patients exhibited a strong inflammatory phenotype as characterized by a stark increase in supernatant proinflammatory cytokine concentrations upon whole blood incubation *ex vivo*. This increase in these very patients, however, was significantly albeit only partially inhibited by a combined triple TLR blockade, as established in robust dose-response studies.

This diversity of immunologic profiles with only a small fraction of patients displaying ongoing proinflammatory cytokine production *ex vivo* despite sampling all patients within 24 h of meeting Sepsis-3 criteria and still unblocked parallel inflammatory pathways might explain futility of previous clinical treatments with TLR-inhibitors. Accordingly, proper *ex vivo* assays may be useful in septic individuals before embarking on immunological treatments.

## Data availability statement

The raw data supporting the conclusions of this article will be made available by the authors, without undue reservation.

## Ethics statement

The studies involving humans were approved by Ethik-Kommission, Universitätsklinikum Essen Robert-Koch-Str. 9-11 45147 Essen. The studies were conducted in accordance with the local legislation and institutional requirements. The participants provided their written informed consent to participate in this study.

## Author contributions

WB: Data curation, Formal Analysis, Funding acquisition, Investigation, Visualization, Writing – original draft, Writing – review & editing. AB: Data curation, Formal Analysis, Funding acquisition, Investigation, Writing – review & editing. RM: Methodology, Writing – review & editing. AW: Methodology, Writing – review & editing. JB: Supervision, Writing – review & editing. FH: Resources, Writing – review & editing. CK: Conceptualization, Funding acquisition, Project administration, Supervision, Writing – review & editing. JP: Conceptualization, Funding acquisition, Project administration, Supervision, Writing – original draft, Writing – review & editing.

## Supplementary material

Supplementary Table 1 Spearman’s correlations of baseline concentrations, increase of concentrations during incubation, and effect of TLR-blockers averaged via rank-sums.
