# Supplementary material for: Substantial heterogeneity of inflammatory cytokine production and its inhibition by a triple cocktail of toll-like receptor blockers in early sepsis
Source: Front Immunol. 2023 Oct 5;14:1277033. doi: 10.3389/fimmu.2023.1277033 (PMC10588698; doi:10.3389/fimmu.2023.1277033)
Supplement: Supplementary file 2 [file DataSheet_2.docx]

Supplementary Material

## Supplementary Figures


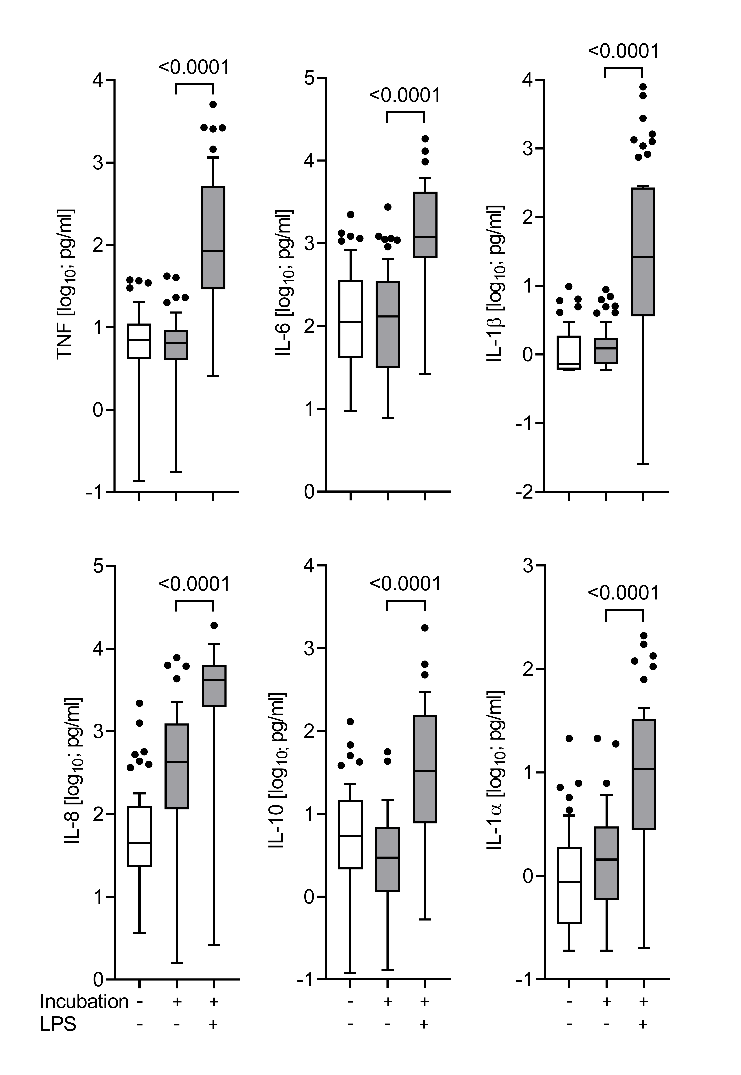


**Supplementary Figure 1** **The cytokine response of whole blood assays to exogenous stimulation by LPS:Re595 extends beyond 6 hours**

Supernatant cytokine concentrations (multiplex ELISA) before and after 22 hours of incubation of whole blood samples from sepsis patients (n=41). LPS: Addition of LPS:Re595 (10 ng/ml ) after 6 h. Tukey boxplots. Wilcoxon matched-pairs tests. Whole blood assays are reactive to TLR stimulation for more than 6 hours.


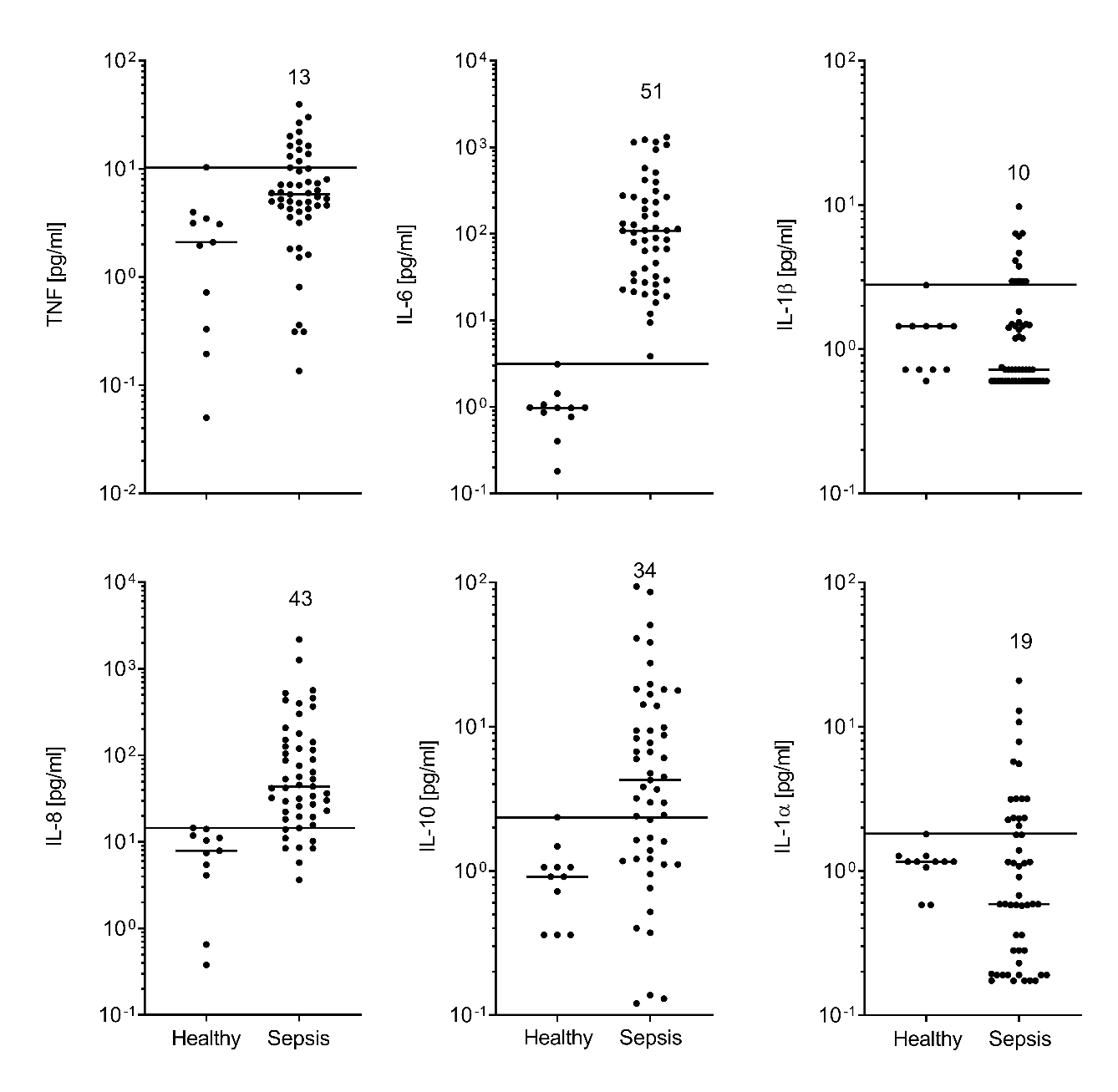


**Supplementary Figure 2** **Increased cytokine concentrations of sepsis patients compared to healthy volunteers.**

**A** Serum concentrations of six inflammatory mediators (multiplex ELISA) of host plasma at baseline from 51 sepsis patients and 11 healthy volunteers. Single point graphs with medians. The number of sepsis patients with cytokine concentrations beyond the maximum concentration of healthy volunteers (long horizontal line) is denoted above the sepsis patients’ data cloud. The IL-6, IL-8, and IL-10 concentrations of most sepsis patients are beyond the maximum concentrations found in healthy volunteers, while TNF and IL-1α and β show a less clear differentiation. The latter two cytokines are only found in lesser concentrations in both sepsis patients’ and healthy volunteers’ samples while IL-6 allows for a clear cut-off.
